# The nuclear translocation of transketolase inhibits the farnesoid receptor expression by promoting the binding of HDAC3 to FXR promoter in hepatocellular carcinoma cell lines

**DOI:** 10.1038/s41419-020-2225-6

**Published:** 2020-01-16

**Authors:** Minle Li, Xuping Zhang, Ying Lu, Sen Meng, Haoyu Quan, Pingfu Hou, Pan Tong, Dafei Chai, Xiaoge Gao, Junnian Zheng, Xuemei Tong, Jin Bai

**Affiliations:** 1 Cancer Institute, Xuzhou Medical University, 221002 Xuzhou, Jiangsu China; 2 Jiangsu Center for the Collaboration and Innovation of Cancer Biotherapy, Xuzhou Medical University, 221002 Xuzhou, Jiangsu China; 3 Department of Biochemistry and Molecular Cell Biology, Shanghai Key Laboratory for Tumor Microenvironment and Inflammation, Key Laboratory of Cell Differentiation and Apoptosis of Chinese Ministry of Education, Shanghai Jiao Tong University School of Medicine, 200025 Shanghai, China; 4 Affiliated Hospital of Xuzhou Medical University, 221002 Xuzhou, Jiangsu China; 5 Center of Clinical Oncology, Affiliated Hospital of Xuzhou Medical University, 221002 Xuzhou, Jiangsu China

**Keywords:** Cancer, Cancer metabolism, Cell biology, Molecular biology

## Abstract

Transketolase (TKT), which is a metabolic enzyme in the nonoxidative phase of the pentose phosphate pathway (PPP), plays an important role in providing cancer cells with raw materials for macromolecular biosynthesis. The ectopic expression of TKT in hepatocellular carcinoma (HCC) was reported previously. However, the role of TKT in the initiation of liver cancer is still obscure. In our previous study, we found that TKT deficiency protects the liver from DNA damage by increasing levels of ribose 5-phosphate and nucleotides. What’s more interesting is that we found TKT deficiency reduced bile acids and loss of TKT promoted the farnesoid receptor (FXR) expression. We further showed that TKT translocated into the nucleus of HCC cell lines through interacting with the signal transducer and activator of transcription 1 (STAT1), and then the complex inhibited FXR expression by promoting the binding of histone deacetylase 3 (HDAC3) to FXR promoter.

## Introduction

Hepatocellular carcinoma (HCC) is the fifth most common tumor in adults^1^ and is a major cause of cancer death worldwide^2^. Despite continuous improvement in treatment over the past decades, the five-year survival rate of HCC is still less than 10%^3,4^. The lack of effective treatment is one of the main reasons for the high mortality of HCC. Therefore, exploring the mechanisms behind the occurrence of HCC and identifying effective target genes are particularly important for the prevention and treatment of HCC.

Some studies have shown that the metabolism of bile acids in liver is closely related to the occurrence of liver cancer. Bile acids are not only physiological detergent molecules synthesized from cholesterol in the liver, but also they are highly cytotoxic^5^. Under normal physiological conditions, bile acid secretion generates bile flow and promotes hepatobiliary secretion of various endogenous metabolites and xenobiotics to increase cholesterol solubility and decrease bile acid toxicity^6^. However, impaired bile flow can lead to cholestasis, and then accumulation of bile acids in the liver causes hepatic inflammation and injury^7^. Studies have shown that cholestasis is related to fibrosis, cirrhosis, and eventually the development of hepatocellular carcinoma.

The farnesoid receptor (FXR) belongs to the nuclear receptor family and is highly expressed in hepatocytes and intestine^8^. Recent studies have shown that FXR not only participates in the regulation of bile acids^9,10^ but also plays an important role in regulating hepatic fibrosis, cholestasis, and inflammation^11–13^. It has been shown that FXR inhibits the absorption of hepatic bile acid and promotes bile acid secretion by promoting the expression of the hepatic bile acid transporter Bile Salt Export Pump (BSEP), which prevents bile acid accumulation in hepatocytes^14^. Some studies have reported that FXR knockout mice develop spontaneous liver tumors^15,16^.

TKT is one of the metabolic enzymes in the nonoxidative phase of the pentose phosphate pathway, which plays an important role in promoting the rapid proliferation of tumor cells. TKT-null mice die in embryo and disruption of one TKT allele can cause growth retardation^17^. Emerging evidence also showed that in cervical and pancreatic cancer, high expression of TKT provides raw materials for DNA and RNA synthesis in tumor cells by enhancing the activity of PPP^18,19^. The ectopic expression of TKT in HCC has been reported previously^4,20^. However, the roles of TKT in the initiation of liver cancer are still obscure. In our previous study, we generated a liver-specific TKT knockout mouse strain, we found a novel role of TKT in increasing genome instability by limiting de novo nucleotide biosynthesis, and loss of the enzyme significantly reduced liver injury and cancer initiation^21^. In addition, metabolomic analysis showed that TKT deficiency reduced bile acids in livers. We further showed that TKT entered the nucleus with the help of STAT1 to inhibit FXR expression through promoting the binding of HDAC3 to FXR promoter. Taken together, our findings may provide a novel metabolic target for HCC prevention.

## Materials and methods

### Mice

The *TKT*^*fl//fl*^*Alb-cre* mice in C57BL/6 genetic background were kindly provided by Dr. Xuemei Tong at Shanghai Jiao Tong University School of Medicine. Animals were maintained under specific pathogen-free conditions and all experiments were conducted in accordance with the guidelines for animal care at the Shanghai Jiao Tong University School of Medicine. In all animal studies, the sample size is more than three pairs.

### Antibodies and reagents

The specific antibodies used in this study were as follows: anti-TKT (8616, Cell Signaling Technology; Abcam, 112997), anti-STAT1 (14994, Cell Signaling Technology), anti-Tubulin (10004185, Proteintech) and anti-PARP (GTX20833, GeneTex), anti-FXR (ab28480, Abcam; 25055-1-AP, Proteintech).

### Mouse models of liver cancer and analysis

In the DEN-induced HCC model, DEN (25 mg/kg) was injected i.p. into 2-week male mice once. Starting at 4 weeks of age, the mice were fed a high-fat diet until they were sacrificed at 6 or 9 months of age. Their livers were removed and separated into different lobes. Visible tumors were counted and measured.

### Mass spectrometry of bile acids in livers

Liver (30 mg) was removed from WT/KO mouse, was added with pre-cold 80% (vol/vol) methanol to extract metabolites followed by centrifugation at 16,000×*g* for 10 min at 4 °C. The supernatant was dried with N2 at room temperature. The extracts were reconstituted with 100 μL 50% (v/v) methanol solution. UHPLC-MS were performed using an HPLC (Dionex 3000 Ultimate)/ MS/MS (TSQ Vantage, Thermo Scientific). A 5 μL of sample was injected for each analysis. The chromatographic column (100 × 2.1 mm, 1.9 μm, Hypersil Gold) was used for separation at 45 °C. Mobile phase A is water with 10 mM ammonium acetate, and mobile phase B is 100% acetonitrile. The flow rate is 0.4 mL/min and gradient program began with 20% B to 23% B in 3 min, 23% B to 32% B in 5 min, 32% B to 80% B in 6 min, and hold on 80% B for 6 min. The instrument was operated in selected reaction monitoring (SRM) and positive ionization mode. The mass analyzer settings were as following: vaporizer temperature (350 °C), spray voltage (3000 V), capillary temperature (330 °C), sheath gas pressure (40 arb), Aux gas pressure (10 arb), collision gas pressure (mTorr):1.5, Q1 peak width (FWHM): 0.7, Q3 peak width (FWHM): 0.7, cycle time (s): 0.4 s. The SRM parameters of dansyl derivatives of amino acids and IS (d3-proline) are shown in Supplementary Table 1. The results were corrected with internal standard, and each liver sample is tested three times, and the group with the largest deviation is removed, and the average value is calculated.

### Cell culture

The human cell lines SMMC-7721, HepG2, and L-O2 were purchased from the Shanghai Institute of Biochemistry and Cell Biology, Chinese Academy of Science (Shanghai, China). SMMC-7721, HepG2, and L-O2 cells were cultured in DMEM medium containing 10% FBS. All cells were maintained in a 5% CO_2_ atmosphere at 37 °C.

### Western blot analysis

Total protein extract was obtained using RIPA lysis buffer (Sigma, St. Louis, MO, USA). The protein concentration was measured by the BCA Protein Assay Kit (Pierce, Rockford, IL, USA). Samples were subsequently resolved on 7.5 and 15% SDS-PAGE gels. Proteins were transferred to Immuno-Blot PVDF Membranes (Bio-Rad, Hercules, CA, USA) and the membranes were blocked in 5% nonfat milk in Tris buffered saline containing Tween-20. The membrane was incubated with the stated primary antibodies followed by secondary peroxidase labeled anti-rabbit or anti-mouse antibodies (Santa Cruz, CA, USA). The signals were developed using an enhanced chemiluminescent solution (Millipore, Boston, MA, USA).

### RNA isolation, reverse-transcription, and real-time quantitative RT-PCR

Total RNA was extracted with TRIzol (Invitrogen Life Technologies, Carlsbad, CA, USA) according to the manufacturer’s instructions. Total RNA was reverse transcribed into cDNA using the PrimeScriptTM RT Reagent Kit (Takara Bio Inc., Shiga, Japan). The SYBR® green Premix Ex TaqTM kit (Takara Bio Inc., Shiga, Japan) was used for real-time PCR analysis, which was performed using an ABI 7500 Fast Sequence Detector (Applied Biosystems, Carlsbad, CA, USA).

### ChIP assay

ChIP assays were performed according to the protocol of the ChIP assay kit (Upstate Biotechnology). SMMC-7721 cells cultured in a 100 mm dish (approximately 1 × 10^7^) were cross-linked by adding formaldehyde to a final concentration of 1% and incubated at room temperature for 10 min, washed twice with cold PBS containing protease inhibitors, lysed in ChIP lysis buffer, then sonicated to shear the DNA at 4 °C to reduce its average length. The sheared DNA was incubated with antibodies against Flag or IgG. DNA-protein-antibody complexes were incubated with ChIP beads with protein A/G (Merck Millipore). The beads were washed with gradients of salt buffer and eluted in 1% SDS/NaHCO_3_. ChIP DNA was analyzed by qRT-PCR with primers amplifying the putative regions. The primers sequence are presented in Supplementary Table 2.

### Luciferase reporter assay

SMMC-7721 cells were transfected, and 48 h later, the cells were harvested and assayed using a luciferase reporter assay system (Promega, USA) according to the manufacturers’ instructions.

### Coimmunoprecipitation

Cells were freshly lysed in lysis buffer (1 mM EDTA, 40 mM Tris-HCl, pH 8, 100 mM NaCl, 0.5% NP-40, 1% Triton X-100), and incubated with primary antibodies at 4 °C overnight, followed by an additional 2-h incubation with protein A/G-agarose beads (Santa Cruz Biotechnology, USA) at 4 °C. The beads were washed with the lysis buffer and boiled in 2× SDS protein loading buffer. Western blot analysis was performed after immunoprecipitation.

### Statistical analysis

All experiments shown were replicated three times at least. Statistical analysis was performed using Prism 5 (GraphPad Software, San Diego, CA, USA). All reported *P* values were two-sided, and *P* *<* 0.05 was considered to be statistically significant.

## Results

### TKT deficiency reduced bile acids in livers

**Fig. 1:**
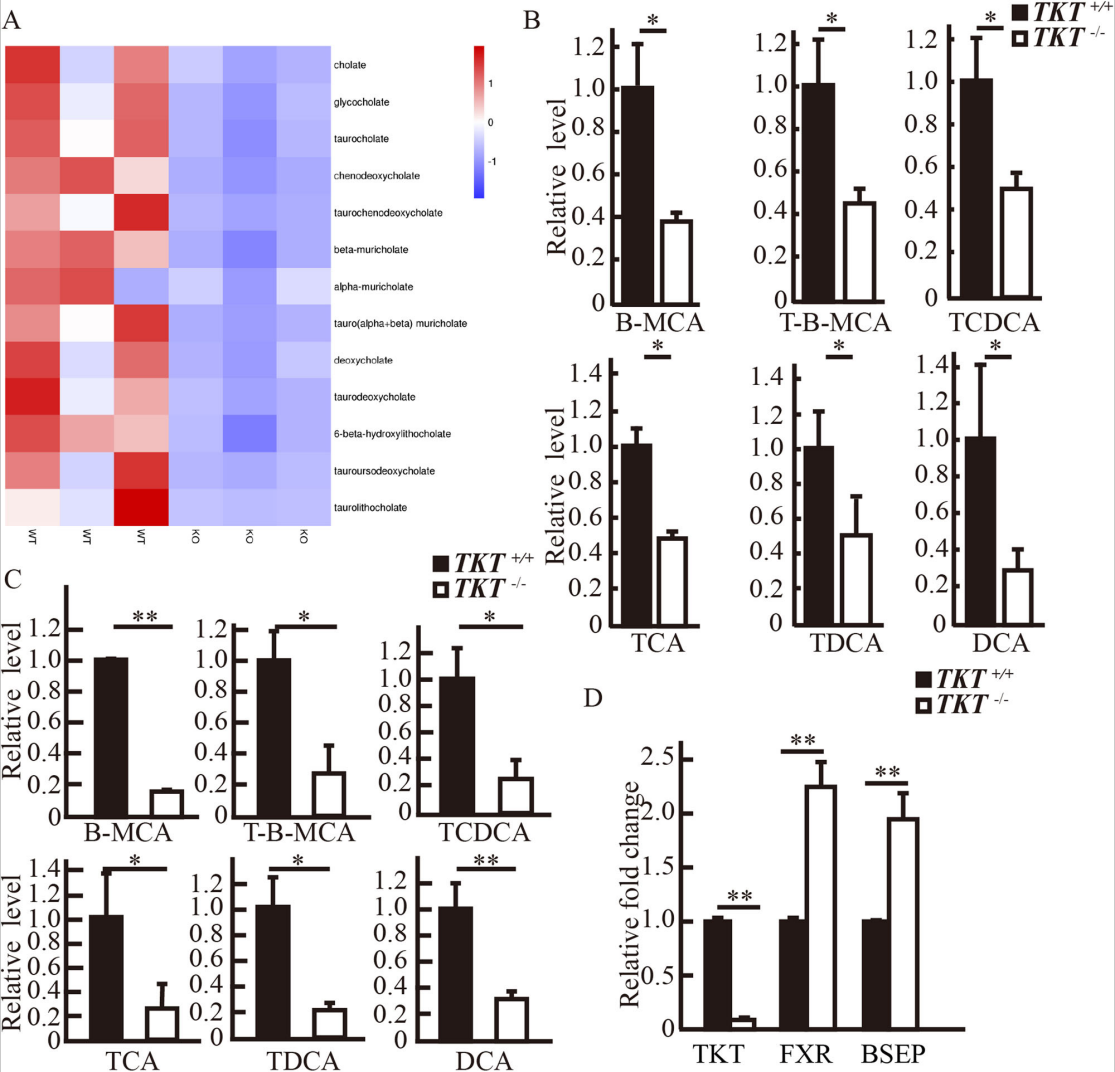
TKT deficiency reduced bile acids in livers. **a** Metabolomic analysis of bile acids in livers from mice at 2 months of age. **b** Mass spectrometric detection of bile acids at 6 months of age in livers from mice following DEN/HFD treatment. **c** Mass spectrometric detection of bile acids at 6 months of age in serum from mice following DEN/HFD treatment. **d** QPCR analysis of TKT, FXR, and BSEP mRNA expression in *TKT*^*+/+*^Alb-cre and *TKT*^*fl/fl*^*Alb-Cre* mice livers. Data are presented as mean ± squared error (SEM), the bar indicates the mean. Statistical significance was calculated using two-tailed unpaired *t*-test, *p < 0.05, **p < 0.01.

To investigate the protective role of TKT deficiency during liver carcinogenesis, we performed metabolomic analysis. Surprisingly, we found that in the livers of *TKT*^*fl/fl*^*Alb-Cre* mice, primary and secondary bile acids were decreased compared with *TKT*^*+/+*^*Alb-cre* mice (Fig. 1a). Then, we performed mass spectrometry in livers and serum from mice following DEN/HFD treatment for 6 months, and the results further confirmed that TKT deficiency reduced bile acids in livers (Fig. 1b, c).

To determine the role of TKT in bile acid metabolism, we detected the level of FXR. QPCR confirmed that loss of TKT could induce the expression of FXR and its target gene BSEP at the mRNA level (Fig. 1d). These results suggested that TKT might regulate FXR expression to affect bile acid level in the liver.

### The expression of FXR is negatively controlled by TKT

**Fig. 2:**
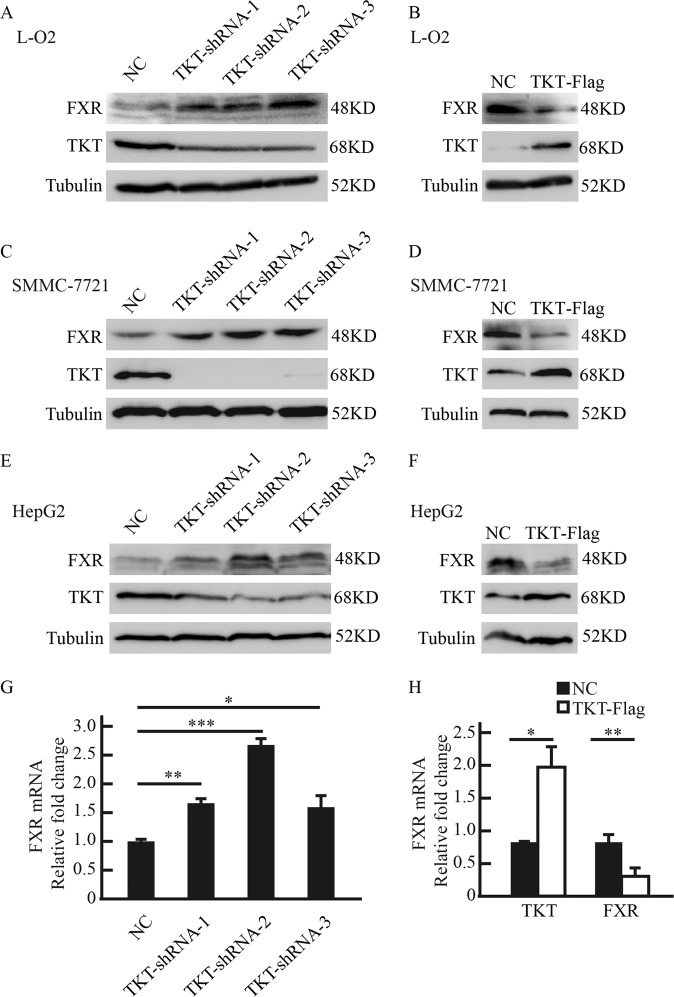
The expression of FXR is negatively controlled by TKT. **a**, **b** Western blot analysis shows that TKT negatively regulates FXR expression in L-O2 cells. **c**, **d** Western blot analysis shows that TKT negatively regulates FXR expression in SMMC-7721 cells. **e**, **f** Western blot analysis shows that TKT negatively regulates FXR expression in HepG2 cells. **g**, **h** QPCR analysis shows that TKT negatively regulates FXR mRNA level in SMMC-7721 cells. Data are presented as mean ± squared error (SEM), the bar indicates the mean. Statistical significance was calculated using two-tailed unpaired *t*-test, *p < 0.05, **p < 0.01, ***p < 0.001.

To determine the relationship between TKT and FXR, we changed TKT expression in normal liver cells L-O2 and two different HCC cell lines, SMMC-7721 and HepG2. Three independent shRNA sequences (TKT-shRNA-1, TKT-shRNA-2, and TKT-shRNA-3) profoundly suppressed TKT expression in L-O2, SMMC-7721 and HepG2. We found that knockdown of TKT increased FXR protein and mRNA levels (Fig. 2a, c, e, and g). In contrast, the overexpression of TKT suppressed FXR production in L-O2, SMMC-7721, and HepG2 (Fig. 2b, d, f and h). These results indicated that TKT regulated FXR expression negatively.

### TKT translocated into the nucleus and regulated the activity of the FXR promoter

**Fig. 3:**
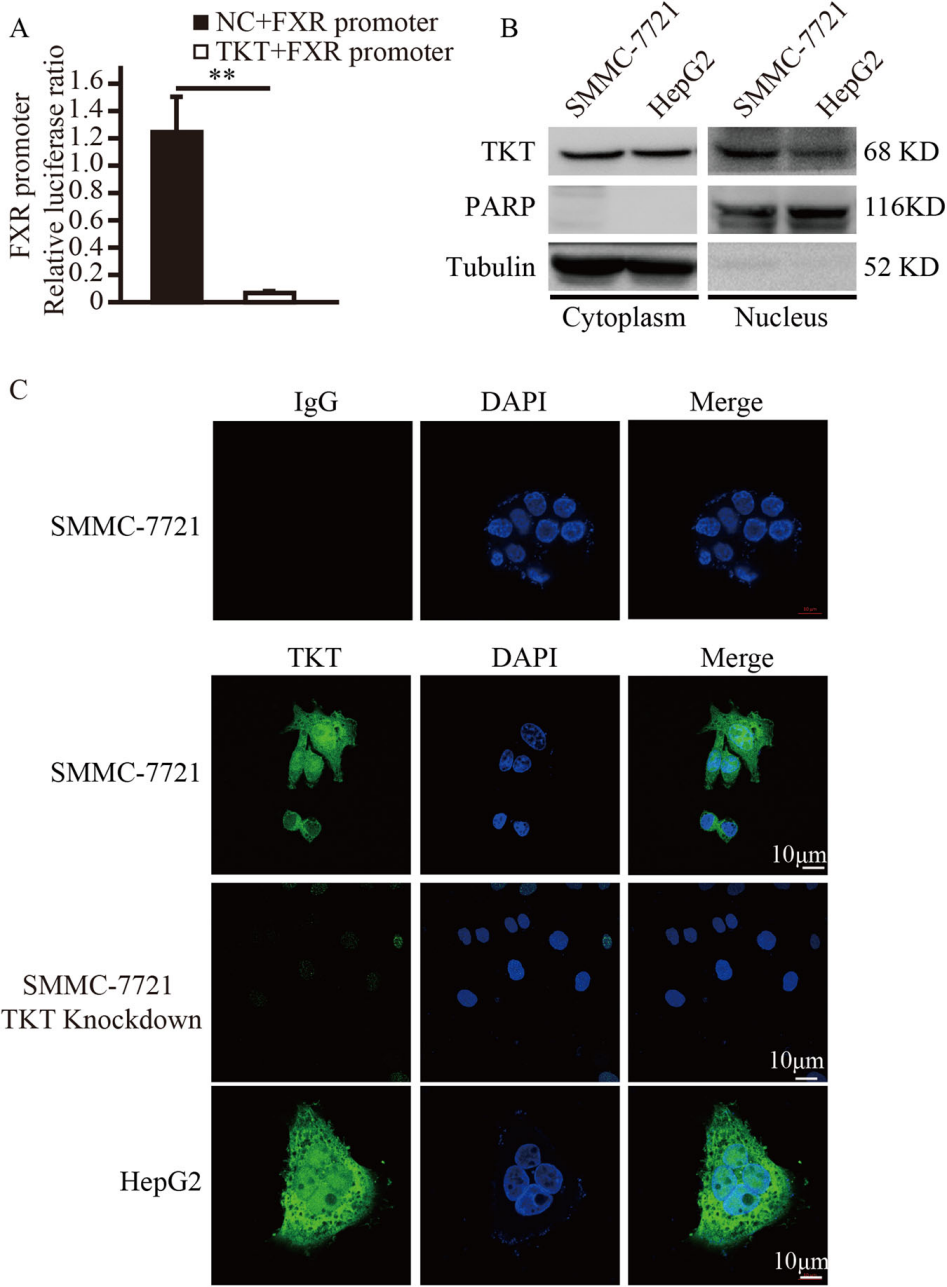
TKT translocated into the nucleus and regulated the activity of the FXR promoter. **a** Luciferase activity analysis of the 2.5 kb FXR promoter at 48 h after the FXR promoter plasmid and empty vector or TKT-Flag expression plasmids were transfected into SMMC-7721 cells, Data are presented as mean ± squared error (SEM), the bar indicates the mean. Statistical significance was calculated using two-tailed unpaired *t*-test, *p < 0.05. **b** Nuclear and cytosolic fractionation analysis shows that TKT are localized in both the cytoplasm and nucleus of SMMC-7721 cells. Tubulin and PARP served as loading controls for the cytosolic and nuclear fraction, respectively. Every sample we loaded two lanes. **c** Immunofluorescent staining of endogenous TKT in SMMC-7721, SMMC-7721 cells with TKT shRNA and HepG2 cells.

Given that TKT negatively regulated FXR expression, we hypothesized that TKT may translocate into the nucleus and regulate the activity of the FXR promoter. To verify our hypothesis, a luciferase reporter assay was conducted in SMMC-7721 cells. We found that overexpression of TKT inhibited the activity of the FXR promoter (Fig. 3a). This result indicates that TKT might be located in the nucleus. Then, we analyzed endogenous TKT subcellular localization using both cell fraction analysis and immunofluorescent staining. Subcellular fractionation showed that TKT was present in both the cytosolic and nuclear fractions (Fig. 3b). Immunofluorescent staining using an anti-TKT antibody confirmed that TKT could be located in the nucleus (Fig. 3c), and immunofluorescent staining using an anti-Flag antibody confirmed that overexpressed TKT could be located in the nucleus (Supplementary Fig. S1). Therefore, TKT can be present in the nucleus where it may regulate the FXR promoter.

### TKT translocated into the nucleus through interacting with STAT1

**Fig. 4:**
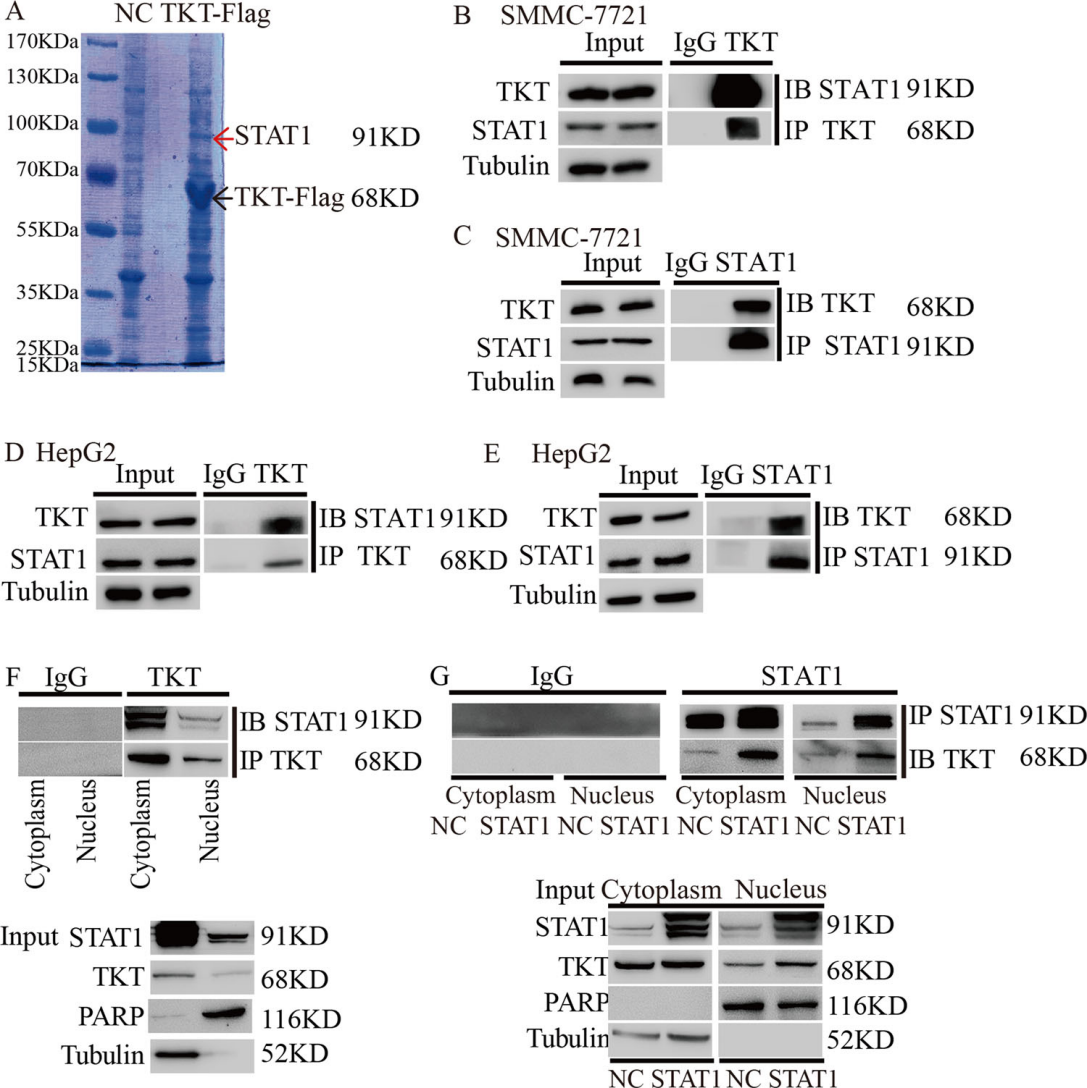
TKT translocated into the nucleus through interacting with STAT1. **a** Silver-staining analysis of SMMC-7721 cell lysates immunoprecipitated using the anti-Flag antibody and normal rabbit IgG (NC). **b**, **c** Endogenous TKT coimmunoprecipitates with STAT1 in SMMC-7721 cells. **d**, **e** Endogenous TKT coimmunoprecipitates with STAT1 in HepG2 cells. **f** Endogenous TKT coimmunoprecipitates with STAT1 from the cytoplasm and nucleus of SMMC-7721 cells. **g** Endogenous TKT coimmunoprecipitates with STAT1 from the cytoplasm and nucleus of SMMC-7721 cells with aberrant expression of STAT1.

Next, we studied how does TKT translocate into the nucleus. We found that TKT had no obvious nuclear location sequence, so we suspected that TKT needs the help of other proteins to enter the nucleus. To find the cofactor, we immunoprecipitated SMMC-7721 cell lysates using the Flag antibody and IgG followed by SDS-PAGE and mass spectrometry analysis. Among the TKT immunoprecipitates, we focused on STAT1 because it can enter the nucleus (Fig. 4a). Then, we verified the interaction between STAT1 and TKT using coimmunoprecipitation. We confirmed that TKT coimmunoprecipitated with STAT1 in SMMC-7721 and HepG2 cells (Fig. 4b–e). Furthermore, coimmunoprecipitation results showed that TKT interacted with STAT1 simultaneously in the cytoplasm and nucleus (Fig. 4f). In addition, STAT1 overexpression increased the interaction in the nucleus (Fig. 4g).

### STAT1 affected TKT nuclear localization and regulation of FXR promoter activity

**Fig. 5:**
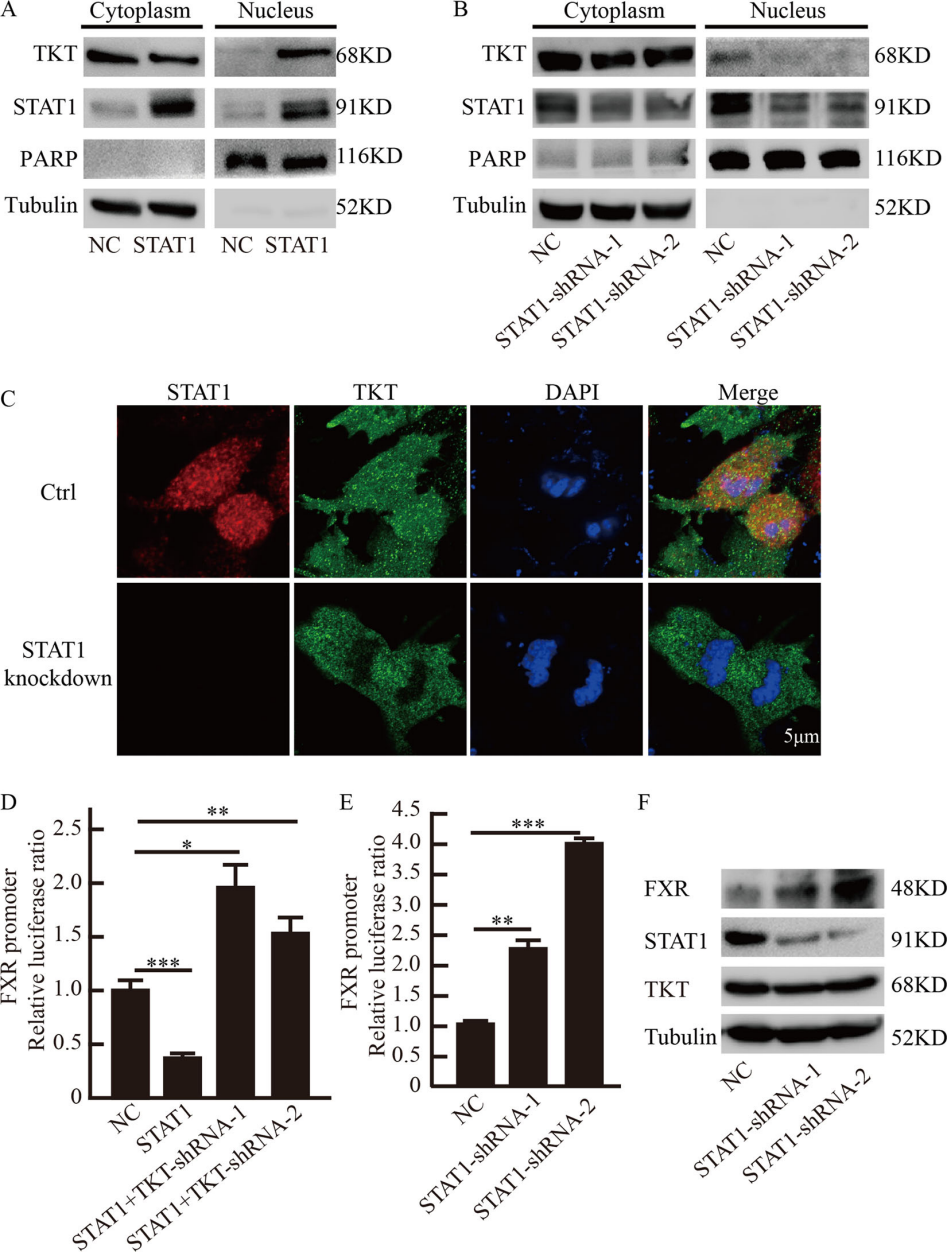
STAT1 affected TKT nuclear localization and regulation of FXR promoter activity. **a** Nuclear and cytosolic fractionation analysis for endogenous TKT in SMMC-7721 cells with aberrant expression of STAT1. **b** Nuclear and cytosolic fractionation analysis for endogenous TKT in SMMC-7721 cells with STAT1 shRNA. **c** Immunofluorescent localization of TKT in SMMC-7721 cells with knockdown STAT1. **d** Luciferase activity analysis of the 2.5 kb FXR promoter at 48 h after the FXR promoter plasmid and empty vector or TKT-shRNA expression plasmids were transfected into the stable overexpression STAT1 cell line SMMC-7721. **e** Luciferase activity analysis of the 2.5 kb FXR promoter at 48 h after the FXR promoter plasmid and empty vector or STAT1-shRNA expression plasmids were transfected into SMMC-7721. Data are presented as mean ± squared error (SEM), the bar indicates the mean. Statistical significance was calculated using two-tailed unpaired *t*-test, *p < 0.05, **p < 0.01, ***p < 0.001. **f** Western blot analysis of FXR, STAT1, and TKT expression after STAT1-shRNA expression plasmids were transfected into SMMC-7721.

To observe whether STAT1 affects TKT nuclear localization, we ectopically expressed STAT1 in SMMC-7721 cells and analyzed TKT subcellular localization using cell fractionation analysis. We found that overexpression of STAT1 promoted TKT nuclear localization (Fig. 5a), and STAT1 shRNA-transfected SMMC-7721 cells had a reduced amount of TKT protein in the nuclear fractions **(**Fig. 5b**)**, immunofluorescent staining further confirmed that STAT1 affected TKT nuclear localization (Fig. 5c and Supplementary Fig. S2).

We then analyzed whether TKT-STAT1 affects the activity of the FXR promoter. We transiently transfected the stable overexpression STAT1 cell line SMMC-7721 with the 2.5 kb FXR promoter linked to luciferase and analyzed luciferase activity 48 h later. The results showed that STAT1 inhibited the activity of the FXR promoter, but interestingly, luciferase activity was increased when the stable cell lines were transfected with TKT shRNA (Fig. 5d), and knockdown of STAT1 promoted luciferase activity in SMMC-7721 (Fig. 5e). Western blot further confirmed that STAT1 knockdown promoted FXR expression (Fig. 5f). These results suggested that STAT1 affected TKT nuclear localization and the regulation of TKT to FXR promoter.

### TKT inhibited FXR promoter activity by promoting the binding of HDAC3 to FXR promoter

**Fig. 6:**
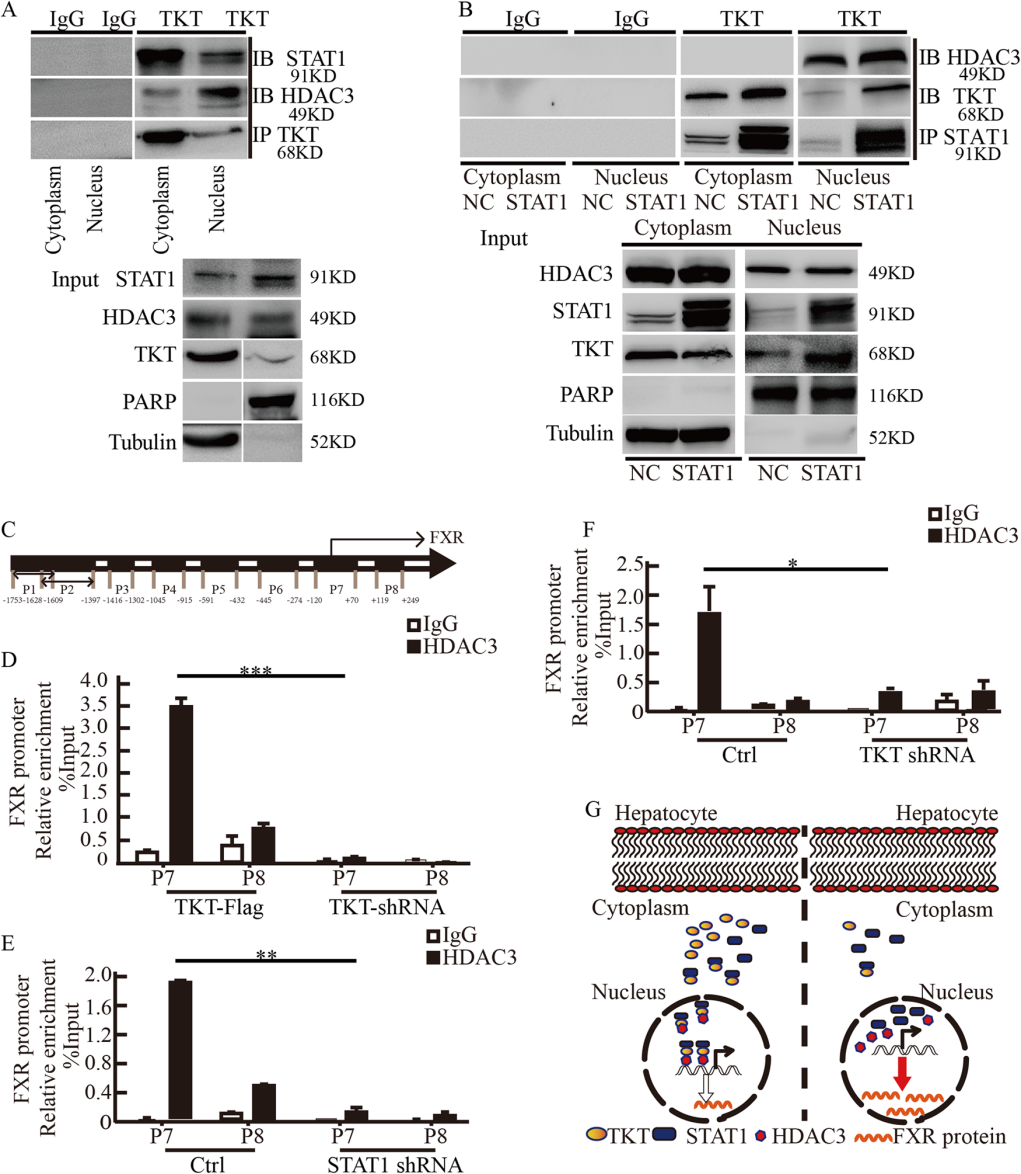
TKT-STAT1 complex promotes the binding of HDAC3 to FXR promoter and inhibits FXR promoter activity. **a** Endogenous TKT coimmunoprecipitates with HDAC3 and STAT1 from the cytoplasm and nucleus of SMMC-7721 cells. **b** Endogenous TKT coimmunoprecipitates with HDAC3 and STAT1 from the nucleus and cytoplasm of SMMC-7721 cells ectopically expressing STAT1. **c** We designed potential binding regions in the promoter of FXR, denoted as P1, P2, P3, P4, P5, P6, P7, and P8. **d** ChIP analysis of SMMC-7721 cells using an anti-HDAC3 antibody or nonspecific IgG shows that the binding capacity of HDAC3 for FXR promoter was decreased when TKT was knocked down in SMMC-7721 cells. **e** ChIP analysis of SMMC-7721 cells using an anti-HDAC3 antibody or nonspecific IgG shows that the binding capacity of HDAC3 for FXR promoter was decreased when STAT1 was knockdown in SMMC-7721 cells. **f** ChIP analysis of SMMC-7721 cells using an anti-HDAC3 antibody or nonspecific IgG shows that the binding capacity of HDAC3 for FXR promoter was decreased when TKT was knockdown in SMMC-7721 cells. Data are presented as mean ± squared error (SEM), the bar indicates the mean. Statistical significance was calculated using two-tailed unpaired *t*-test, *p < 0.05, **p < 0.01, ***p < 0.001. **g** Schematic model of the role of TKT in regulating FXR expression.

Given that TKT nuclear localization needed the help of STAT1, we next investigated how TKT regulates FXR promoter activity in the nucleus. Histone acetylation is one of the most important components of epigenetics. Histone deacetylases (HDACs) have emerged as major enzymes in the epigenetic regulation of gene expression by catalyzing the removal of acetyl groups, acetylation is one of the significant epigenetic markers associated with histones in the enhancers and promoters of genes, leading to chromatin remodeling and alterations in gene expression^22^. By using mass spectrometry analysis, we found that TKT could interact with HDAC3. We tried to verify the interaction between TKT, STAT1, and HDAC3 using coimmunoprecipitation (Fig. 6a).

After confirming that STAT1 affects TKT nuclear localization, we then investigated whether STAT1 affected the interaction between TKT and HDAC3 in the nucleus. Cell fractionation analysis showed that STAT1 overexpression increased the interaction of TKT and HDAC3 in the nucleus (Fig. 6b).

We postulated that TKT inhibited FXR expression through promoting the binding of HDAC3 to FXR promoter. We designed potential binding regions in the promoter of FXR, denoted as P1, P2, P3, P4, P5, P6, P7, and P8 (Fig. 6c). To determine whether TKT affected HDAC3’s recruitment to the potential binding region in the FXR promoter, a chromatin immunoprecipitation (ChIP) assay was performed in SMMC-7721 TKT knockdown and overexpressing cells. ChIP-quantitative PCR results showed that HDAC3 mainly binds to the P7-binding regions of the FXR promoter, and TKT promotes this binding (Fig. 6d). Furthermore, both STAT1 silencing and TKT silencing reduced the binding of HDAC3 to FXR promoter (Fig. 6e, f), western blot analysis showed that STAT1 and TKT are indeed knocked down (Supplementary Fig. S3). These results suggested that TKT inhibited FXR promoter activity by promoting the binding of HDAC3 to FXR promoter.

## Discussion

Here we have identified a novel role of TKT in regulating bile acid in liver. Bile acids is one of the toxins to cause chronic inflammation and cell death^23^. Studies have indicated deoxycholic acid DCA can cause DNA damage, activation of hepatic stellate cells and the secretion of inflammatory tumor promoting factor. Intrahepatic cholestasis induces excessive fat accumulation, which could cause hepatocyte damage and inflammation, then promotes HCC^24^. Lithocholic acid (LCA) can stimulate the malignant proliferation of colon and breast cancer cells^25,26^. Therefore, bile acids are tumor promoters and are involved in hepatocarcinogenesis. However, bile acid metabolism-target cancer therapy is still in its infancy.

FXR acts as a multifunctional cell protector in the liver through regulating the homeostasis of bile acids critically. FXR overexpression has been demonstrated to repress cancer cell proliferation^27,28^. It is has been shown that mice that have high levels of FXR do not develop liver cancer after treatment with DEN^29^. Therefore, FXR is a tumor suppressor.

Metabolic enzymes play important roles in tumorigenesis. TKT catalyzes several key reactions of the nonoxidative phase of PPP, allowing cells to adapt to a variety of metabolic needs under different conditions^30^. It has been demonstrated that TKT promoting breast cancer metastasis through regulating the metabolic switch^31^. In several human cancers, such as HCC and metastatic peritoneal implants of ovarian cancer, TKT expression is increased significantly^4,18^. Furthermore, TKT levels is closely related to chemotherapeutic resistance of tumor^32–34^. Of note, it has been shown that long noncoding RNA TSLNC8 blocks tumor growth by reducing the interaction between TKT and STAT3 and inhibiting IL-6/STAT3 signaling pathway^35^. Therefore, TKT is a key factor involved in tumorigenesis.

Previous studies have focused on TKT as a metabolic enzyme regulating the PPP pathway to affect tumor cell proliferation. In our study, we have made a metabolomics analysis of livers of WT and *TKT*^*fl/fl*^*Alb-cre* mice. The results showed that compared with littermates, lacking of TKT altered bile acid metabolism, primary and second bile acid were decreased. This is an interesting phenomenon, which means that TKT may affect the development of HCC through regulating intrahepatic bile acids. During liver injury induced by DEN, cholestasis exacerbate liver inflammation, however, mice that have high levels of FXR expression do not develop liver cancer after treatment with DEN^29^.

In the current study, TKT deficiency in hepatocytes alleviated bile acids level in liver. Accordingly, our results showed that TKT was present not only in the cytosolic fraction but also in the nuclear fraction, and that TKT needs to interact with STAT1 to translocate into the nucleus, where with the complex promotes the binding of HDAC3 to FXR promoter, eventually inhibiting the expression of FXR (Fig. 6g). Taken together, we identified a novel role of TKT in regulating intrahepatic bile acid. Indeed, reduced cholestasis in hepatocytes offers an opportunity of HCC therapy. Therefore, targeting the metabolic enzyme TKT is an attractive strategy for bile acid metabolism in livers and cancer therapy.

## Supplementary information

Supplementary Figure Legends

supplementary materials

Figure S1

Figure S2

Figure S3
