# Supplementary material for: The nuclear translocation of transketolase inhibits the farnesoid receptor expression by promoting the binding of HDAC3 to FXR promoter in hepatocellular carcinoma cell lines
Source: Cell Death Dis. 2020 Jan 16;11(1):31. doi: 10.1038/s41419-020-2225-6 (PMC6965636; doi:10.1038/s41419-020-2225-6)
Supplement: Supplementary file 1 — Supplementary Figure Legends [file 41419_2020_2225_MOESM1_ESM.docx]

Supplementary figure legends

Figure S1: Immunofluorescent staining of exogenous TKT with flag-tagged in SMMC-7721 cells.

Figure S2: Immunofluorescent localization of TKT in SMMC-7721 cells with overexpression STAT1.

Figure S3: Western blot analysis verifies that STAT1 and TKT were knockdown in SMMC-7721 cells.
