# Supplementary material for: The nuclear translocation of transketolase inhibits the farnesoid receptor expression by promoting the binding of HDAC3 to FXR promoter in hepatocellular carcinoma cell lines
Source: Cell Death Dis. 2020 Jan 16;11(1):31. doi: 10.1038/s41419-020-2225-6 (PMC6965636; doi:10.1038/s41419-020-2225-6)
Supplement: Supplementary file 2 — supplementary materials [file 41419_2020_2225_MOESM2_ESM.docx]

**The nuclear translocation of transketolase regulates the farnesoid receptor expression by promoting the binding of HDAC3 to FXR promoter in hepatocellular carcinoma cell lines**

Minle Li^1, 2 *^, Xuping Zhang^1, 2*^, Ying Lu^4*^, Sen Meng^1, 2^, Haoyu Quan^1, 2^, Pingfu Hou^1, 2^, Pan Tong^5^, Dafei Chai^1, 2^, Xiaoge Gao^1, 2^, Junnian Zheng^1, 2, 3#^, Xuemei Tong^4#^, Jin Bai^1, 2#^

**Supplementary Table1: SRM parameters of bile acids**

| Compound | Retention  Time(min) | Q1 MS  (m/z) | Q3 MS  (m/z) | CE  (V) | S-lens  (V) |
| --- | --- | --- | --- | --- | --- |
| LCA | 13.10 | 375.3 | 375.3 | 5 | 157 |
| LCA-d4 | 13.10 | 379.3 | 379.3 | 5 | 125 |
| UDCA/HDCA/CDCA/DCA | 8.60/9.15/11.07/11.20 | 391.3 | 391.3 | 5 | 150 |
| DCA-d4 | 11.20 | 395.3 | 395.3 | 5 | 129 |
| Alpha-MCA/beta-MCA/CA | 5.76/6.09/8.64 | 407.3 | 407.3 | 5 | 160 |
| CA-d4 | 8.64 | 411.3 | 411.3 | 5 | 134 |
| GLCA | 11.58 | 432.3 | 73.8 | 40 | 140 |
| GUDCA/GHDCA/GCDCA/GDCA | 7.50/7.74/10.26/10.52 | 448.3 | 73.8 | 41 | 136 |
| GUDCA-d4 | 7.47 | 452.3 | 73.8 | 38 | 146 |
| GdhCA | 3.21 | 458.3 | 73.8 | 39 | 147 |
| GCA | 8.07 | 464.3 | 73.8 | 42 | 160 |
| GCA-d4 | 8.07 | 468.3 | 73.8 | 40 | 150 |
| TLCA | 11.66 | 482.3 | 80.0 | 64 | 203 |
| TUDCA/THDCA/TCDCA/TDCA | 8.05/8.28/10.49/10.72 | 498.3 | 80.0 | 66 | 193 |
| TdhCA | 3.70 | 508.3 | 508.3 | 5 | 230 |
| TalphaMCA/TbetaMCA/TCA | 5.55/5.70/8.55 | 514.3 | 124.0 | 52 | 183 |

**Supplementary Table 2: CHIP primers sequence**

| No. | Sequence |
| --- | --- |
| P1-For | AGCCTAAGGTCTAAGGTCAC |
| P1-Rev | TGGATGATAAGAGCCTGTAC |
| P2-For | GTACAGGCTCTTATCATCCA |
| P2-Rev | TCCTACAGGCTCCTACTTCC |
| P3-For | CTGACCTTGTGACGGGAAAC |
| P3-Rev | CAGGCATGGTAGCAGACTTG |
| P4-For | ATAAGGTCAGAGGATTTAAGGGAC |
| P4-Rev | CAGGCGTGGAACAGAAGCAG |
| P5-For | GCCACAAATTATTGGGTGAT |
| P5-Rev | ACAGCCTTGAGCAAGACACT |
| P6-For | TTGCTCAAGGCTGTGACTAAA |
| P6-Rev | AACCCACATTGTTCTTCTCCTA |
| P7-For | AGGGAAGATGACAGTTTGGT |
| P7-Rev | ATAGAAAGGAACCTTGGGAG |
| P8-For | ATGAGGCACAGAGCCAAGGA |
| P8-Rev | CCAATTTCGCATTAGGATAAGT |
