# Supplementary figures and images for: The nuclear translocation of transketolase inhibits the farnesoid receptor expression by promoting the binding of HDAC3 to FXR promoter in hepatocellular carcinoma cell lines
Source: Cell Death Dis. 2020 Jan 16;11(1):31. doi: 10.1038/s41419-020-2225-6 (PMC6965636; doi:10.1038/s41419-020-2225-6)

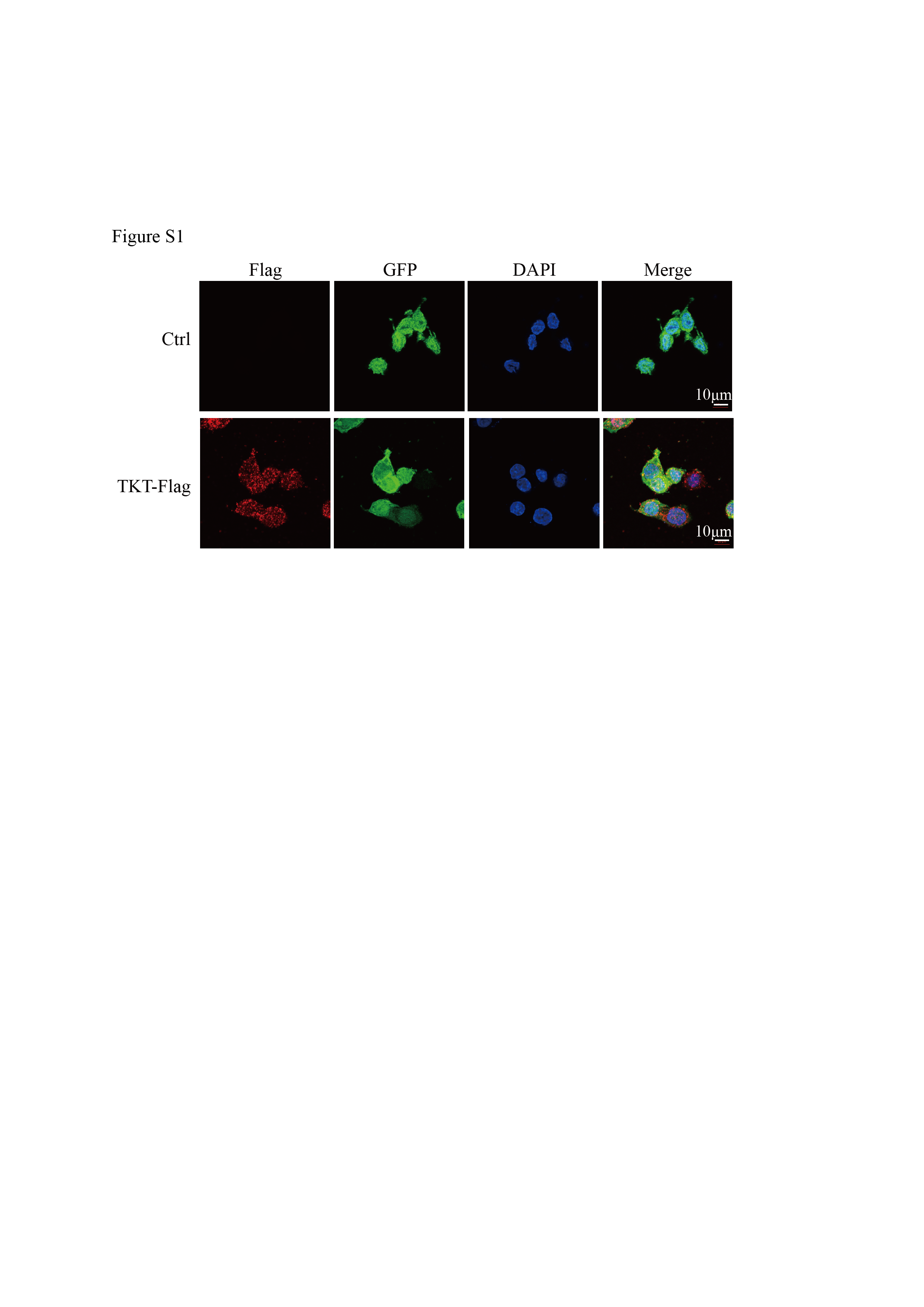

Supplement: Supplementary file 3 — Figure S1 [file 41419_2020_2225_MOESM3_ESM.png]

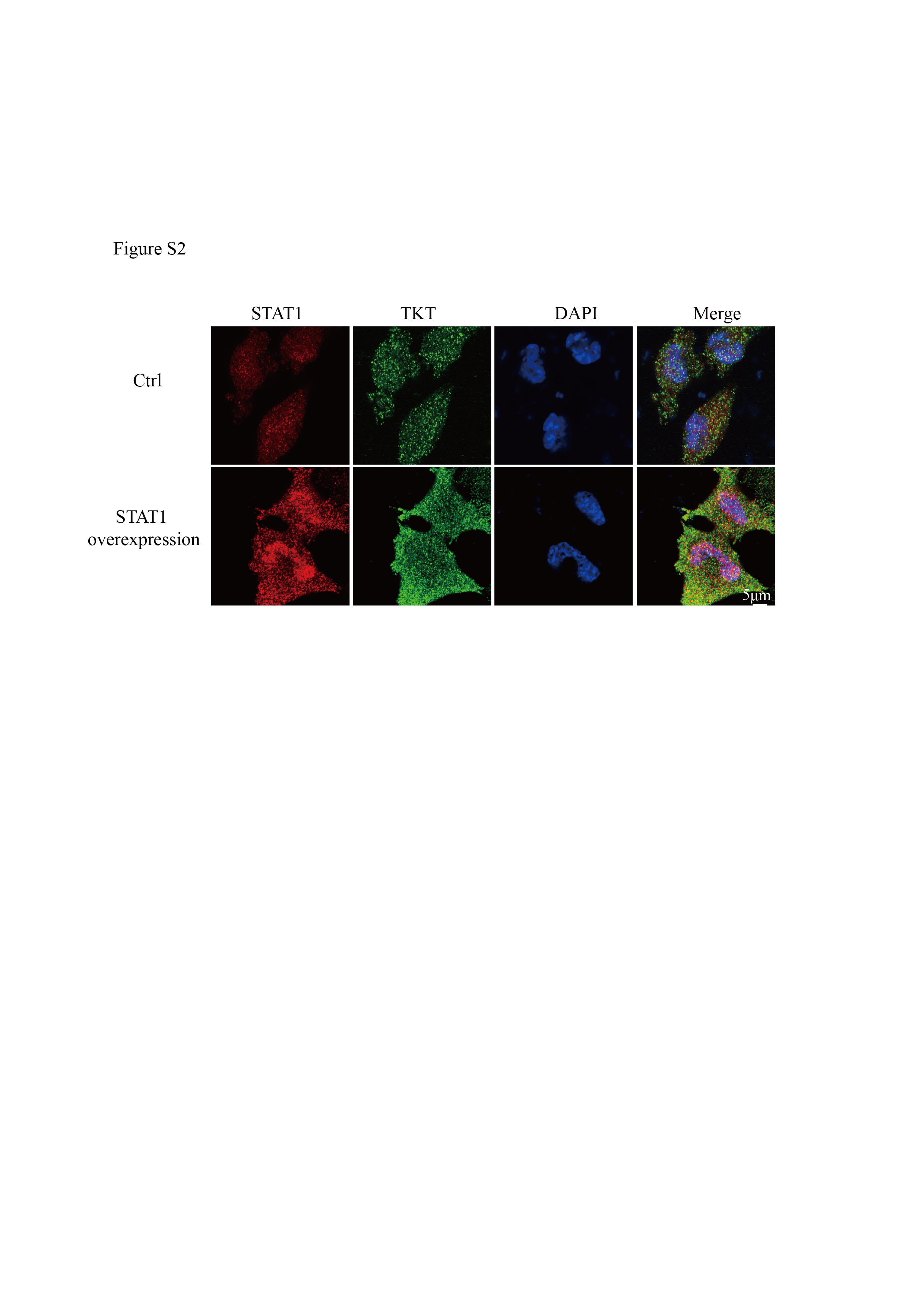

Supplement: Supplementary file 4 — Figure S2 [file 41419_2020_2225_MOESM4_ESM.png]

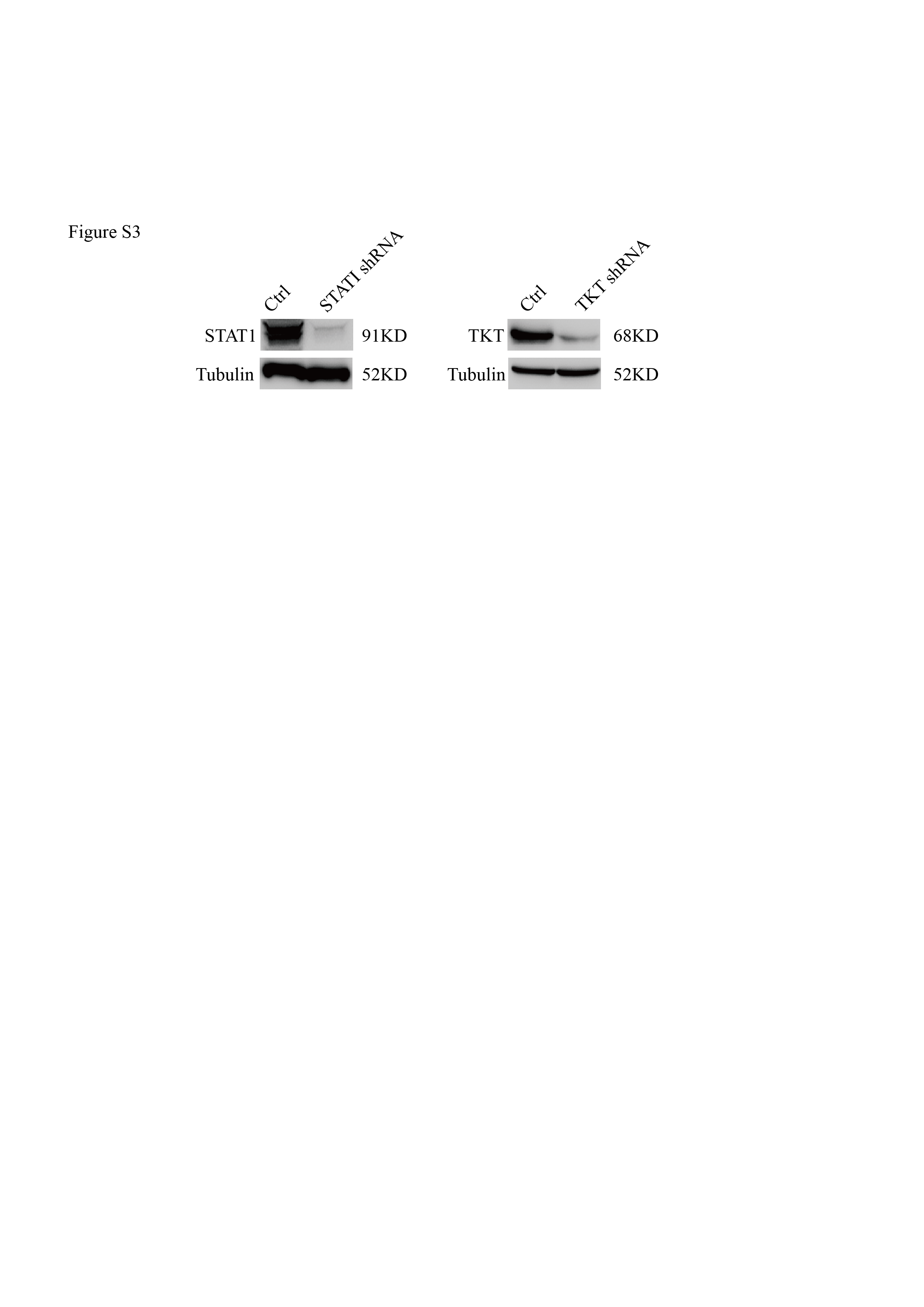

Supplement: Supplementary file 5 — Figure S3 [file 41419_2020_2225_MOESM5_ESM.png]
